# The regulation of hypoxia-related lncRNAs in hepatocellular carcinoma

**DOI:** 10.1007/s12672-024-01002-3

**Published:** 2024-05-07

**Authors:** Xuejing Wang, Xiaojun Wang

**Affiliations:** Department of Integrated Traditional Chinese and Western Medicine, Beijing Youan Hospital, Capital Medical University, Beijing, 100069 China

**Keywords:** Hepatocellular carcinoma, Hypoxia, lncRNA, Metastasis, Prognosis, Biomarkers

## Abstract

Hepatocellular carcinoma (HCC) is still a public health disease with its high prevalence and morbidity. Short of early diagnosis biomarkers and effective therapy, the treatment of HCC patients hasn’t achieved ideal effect. Hypoxia is a hallmark of HCC, which is mainly induced by imbalance of tumor cell proliferation and insufficient supply of oxygen. Recently, amounting evidence suggested lncRNAs, especially hypoxia-related lncRNAs play a pivotal role in regulating HCC. Hypoxia-related lncRNAs are involved in altering glucose metabolism, maintaining of cancer stem cell-like properties (CSCs), cell apotosis, proliferation and immune escape, which all contribute to the poor prognosis of HCC patients. The novel identified hypoxia-related lncRNAs could be the potential target or biomarkers of HCC, which are beneficial to the clinical treatment. Herein, we summarized currently reported hypoxia-related lncRNAs and their related mechanisms, providing potential application and future perspective of hypoxia-related lncRNAs as a potential therapeutic target.

## Introduction

Hepatocellular carcinoma (HCC) ranks as the fourth leading cause of cancer-related mortality worldwide [1, 2]. HCC is characterized by its high prevalence, strong resistance to treatment, and subsequent poor prognosis. Currently, there is a lack of efficient therapy and early biomarkers for HCC [3–5]. The absence of obvious symptoms in the early stages makes it challenging to achieve a definite diagnosis [6]. Typical symptoms such as indigestion, weight loss, jaundice, and distant thoracic metastasis only emerge when the tumor has locally advanced or metastasized. As a result, over a million new cases of HCC are diagnosed each year, with most patients already in an advanced stage [3]. Furthermore, HCC is characterized by its propensity for metastasis, recurrence, and resistance to therapy. Current approaches such as surgical resection or chemotherapy have not yielded satisfactory results [7, 8]. Moreover, the effectiveness of tyrosine kinase inhibitors (TKIs), including first-line TKIs (sorafenib and lenvatinib) and second-line TKIs (regorafenib and cabozantinib), in improving HCC is limited due to their adverse reactions and high cost [9, 10]. Despite significant efforts dedicated to identifying early-stage HCC, the benefits remain unsatisfactory. One underlying reason for this is the hypoxic microenvironment within HCC [11, 12].

Hypoxia, a common characteristic of cancer, contributes to the angiogenesis, metabolism reprogramming, proliferation, and chemoresistance of HCC [13–15]. Three factors contributed to the occurrence of hypoxia: firstly, it is caused by tumor growing and abnormal angiogenesis resulting in insufficient oxygen and nutrients; secondly, local hypoxia occurs as a result of transcatheter arterial chemoembolization (TACE) procedures; finally, anti-angiogenic therapy exacerbates hypoxia by suppressing neovascularization [16–18]. In contrast to human mammary epithelial cells (HMEC) and normal fibroblast cells, hypoxia promotes the growth of HCC by regulating the expression of hexokinase II and insulin-like growth factor-2 [19]. Furthermore, hypoxia, as a key feature in the microenvironment of HCC, mediates changes in the expression of non-coding RNAs [20, 21]. Therefore, it is crucial to uncover the mechanism by which non-coding RNAs interact with hypoxia microenvironment.

**Fig. 1:**
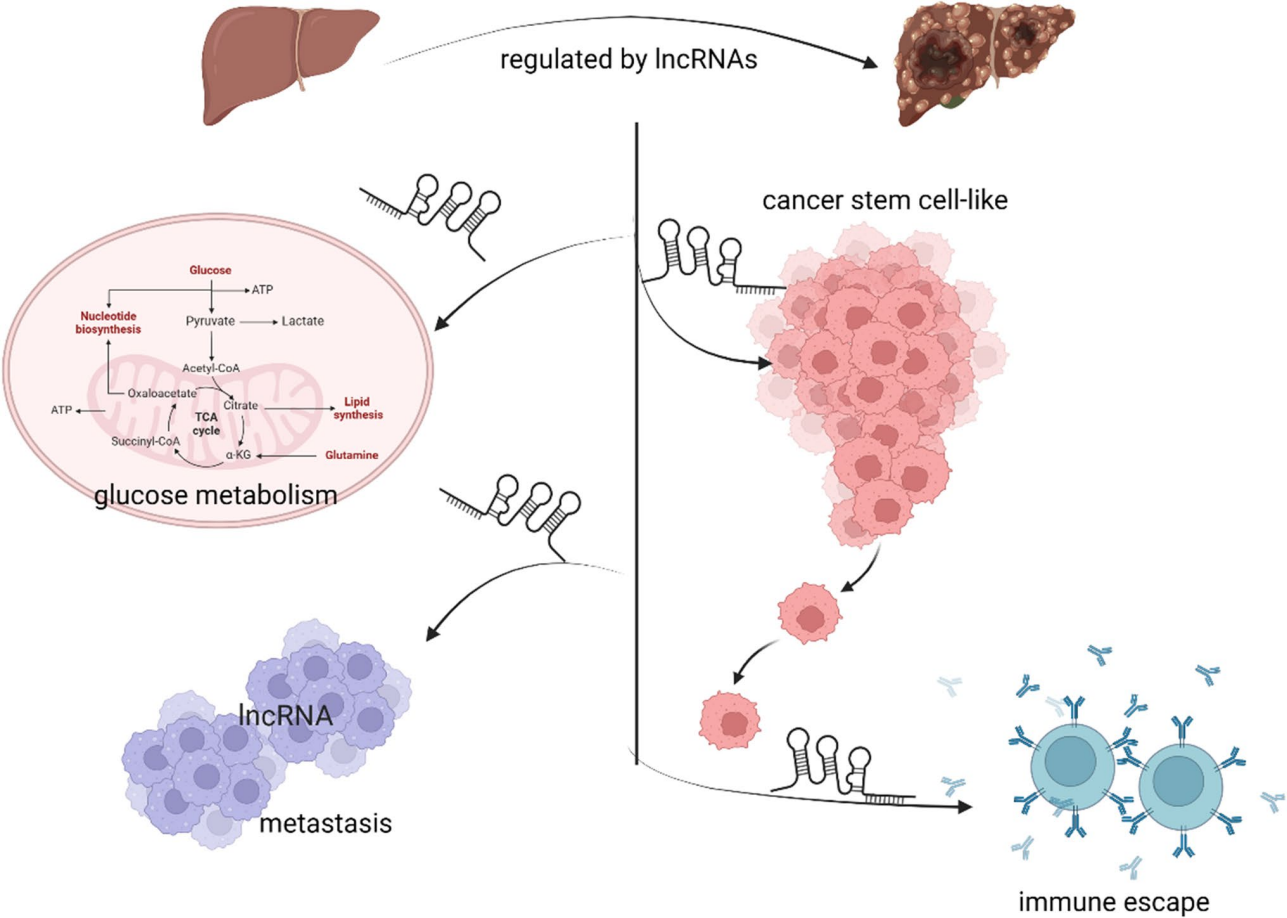
The regulation of lncRNAs during the oncogenesis of HCC. Studies showed that lncRNAs played a pivotal role in regulating glucose metabolism, aggression and metastasis, cancer stem cell-like properties, and immune escape

Long non-coding RNAs (lncRNAs), consisting of more than 200 nucleotides, lack protein-coding function and regulate various tumor biological processes. LncRNA participates in diverse cellular functions: on the one hand, they affect epigenetic regulation by competitively binding with other RNAs or proteins; on the other hand, they mediate the stability of mRNA and protein [20, 22]. A growing body of evidence has demonstrated the pivotal role of lncRNAs in the hypoxia-response process of HCC. It has been reported that lncRNAs are involved in glucose metabolism [23–25], aggression and metastasis [20, 26–28], cancer stem cell-like properties [29], and immune escape [30, 31] (Fig. 1). Furthermore, the release of lncRNAs from the tissue into circulating blood [32] during necrosis or apoptosis suggests that identifying novel and reliable lncRNA biomarkers is crucial for early diagnosis of HCC, providing a non-invasive and safe detection method. This review aims to summarize hypoxia-associated lncRNAs in HCC, explore their related signaling pathways, and provide an overview of current therapeutic advancements.

## Function of hypoxia-associated lncRNAs in HCC

### Hypoxia-associated lncRNAs and metastasis

Metastasis, encompassing tumor cell invasion, migration to target sites, transition into distinct states, and subsequent interaction with local proteins and cells [33–35], is the primary cause of cancer-related death. Despite significant advancements in treatment, the prognosis for HCC remains unsatisfactory due to frequent recurrence or metastasis [36, 37]. Up to now, the concrete mechanisms of metastasis are still elusive; therefore, it is urgent to identify essential genetic regulations that can improve HCC prognosis.

**Fig. 2:**
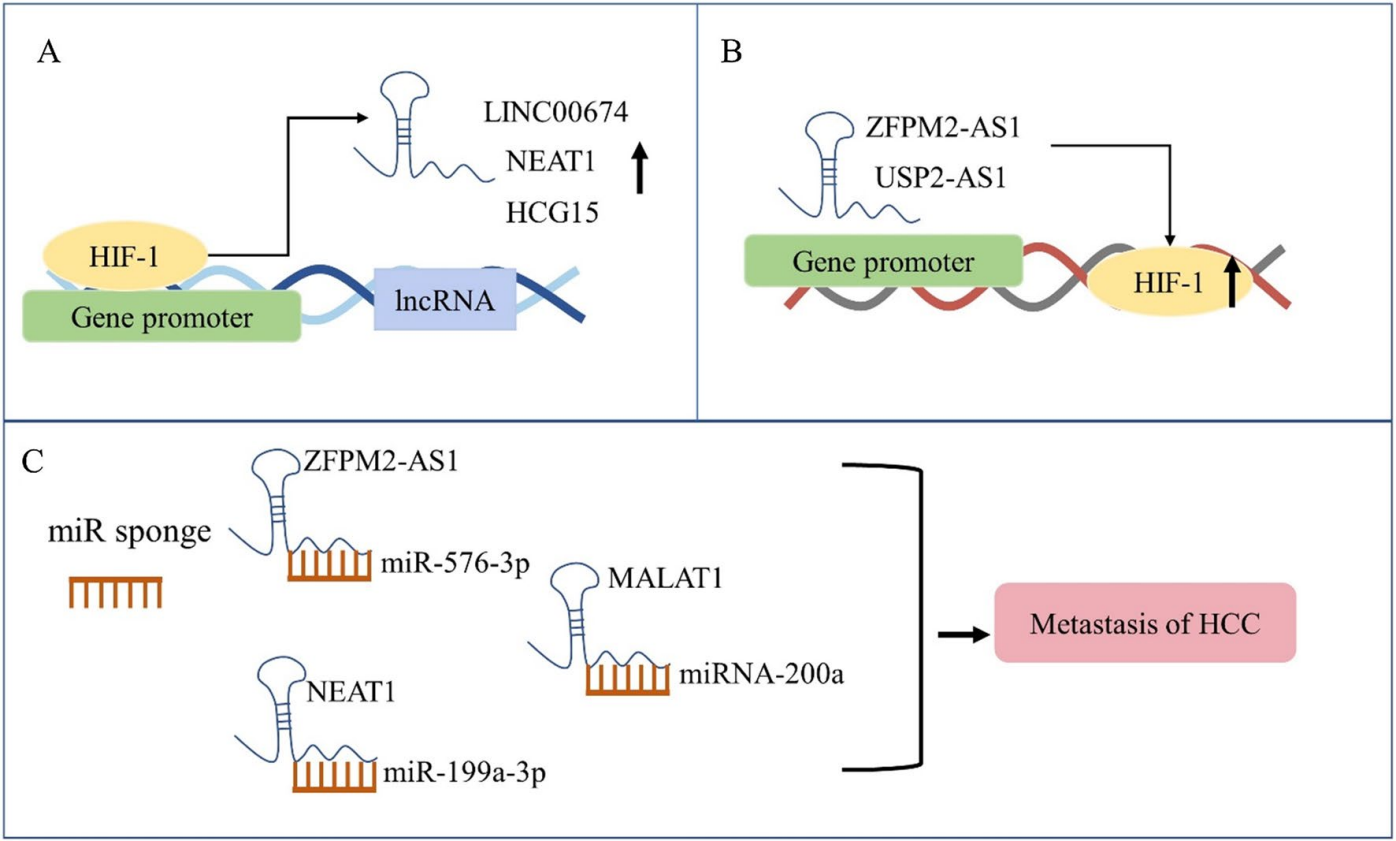
Interaction between HIF-1 and hypoxia-associated lncRNAs in HCC. **A** the expression of lncRNAs LINC00674, NEAT1 and HCG15 were regulated by HIF-1. HIF could bind to the gene promoter of those lncRNAs to increase their expression in HCC; **B** the expression of HIF-1 was mediated by lncRNA ZFPM2-AS1 and USP2-AS1 in turn, indicating their interacted function; **C** excepted the interacted function with HIF-1, those lncRNAs sponged with miR to further promote metastasis of HCC

The proliferation of cancer cells leads to increased oxygen consumption and subsequent hypoxia tumor microenvironment. And hypoxia-inducible factors (HIFs) is responsible for hypoxia response by regulating gene transcription (Fig. 2), in which lncRNAs are included as HIFs target genes [38–40]. LINC00674 was observed to increase in HCC under hypoxic conditions dependent on the occupancy of HIF-1 to HRE of LINC00674 gene promoter. It stimulated the proliferation and metastasis of HCC via activating the NOX1/mTOR signaling pathway. Besides, according to clinical statistical analysis, its expression was positively relevant to the size, stage, and even the poor prognosis of HCC [38]. Similarly, the expression of LncRNA-NEAT1 was also proved upregulated in HCC cell lines under hypoxia, which was also maintained by HIF-1. The researchers speculated that lncRNA-NEAT1 interacted with tumor-suppressive miRNA miR-199a-3p to further sustain the growth of HCC even under a hypoxia environment [41]. Besides, lncRNA MALAT1 sponged microRNA-200a in hypoxic Hep3B cells to affect proliferation, migration, invasion, and apoptosis [42]. Another microarray data analysis determined HLA complex group 15 (HCG15) as the novel hypoxia-responsive lncRNA. It was observed that knocking down the expression of HCG15 blunted the migration, invasion, and proliferation of HCC cells, while upregulation of HCG15 resulted in markedly enhanced proliferation of HCC. What’s more, the expression of HCG15 was also mediated by HIF-1 determined by the downregulation of HIF-1 [20]. Collectively, we can see those lncRNAs are all increased under hypoxia and regulated by HIF-1, which showed that HIF-1 is a crucial upstream control switch and we should focus on its multiple transcription regulation function. Additionally, we also should pay attention to aberrant expression of lncRNA that are potential biomarkers in HCC.

Except for those lncRNAs regulated by HIF-1, some other lncRNAs could regulate the expression of HIF-1 in turn. The significantly increased expression of LncRNA zinc finger protein multitype 2 antisense RNA 1 (ZFPM2-AS1) was confirmed by RT-PCR in HCC cell lines. Further, ZFPM2-AS1 was capable of binding to miR-576-3p and positively regulating the expression of HIF-1α, to promote the proliferation, migration, and invasion of HCC cells [43]. Likewise, lncRNA USP2-AS1 was demonstrated to promote the growth of HCC by stimulating YBX1-mediated HIF1α protein translation under a hypoxia environment [28]. From this perspective, we can see the interplay of lncRNAs and HIF-1, which revealed that their complicated function net, that is, HIF-1 could be either downstream or upstream of lncRNAs.

Therefore, we still need to further investigate the underlying interacted loop between HIF-1 and lncRNAs. In Table 1, we summarized the role of hypoxia-associated lncRNAs in metastasis and progression in HCC, presenting the concrete pathway and cell lines.

**Table 1:** Summarized role of hypoxia-associated lncRNAs of metastasis and progression in HCC

	LncRNAs	Expression	Role/pathway	Outcomes	Cell line	References
Metastasis and progression	LINC00674	Upregulation	Activating the NOX1/mTOR signaling pathway	Facilitating hepatocellular carcinoma progression	Hep3B and MHCC97H	[38]
	NEAT1	Upregulation	Regulating of miR-199a-3p/UCK2	Sustaining the growth of hepatocellular carcinoma	SNU-182 and HUH7	[41]
	MALAT1	Upregulation	Sponging MicroRNA-200a	Regulating hepatocellular carcinoma growth	Huh7, SNU-423, PLC, and Hep3B	[42]
	HCG15	Upregulation	Facilitating hepatocellular carcinoma cell proliferation and invasio	Enhancing ZNF641 transcription	Hep3B and Huh7	[42]
	ZFPM2-AS1	Upregulation	Promoting the proliferation, migration, and invasion of hepatocellular carcinoma	Regulating the miR-576-3p/HIF-1α axis	Huh7, HCCLM3, Hep3B, SMMC-7721, and QSG-7701	[43]
	USP2-AS1	Upregulation	Promoting hepatocellular carcinoma growth	Enhancing YBX1-mediated HIF1α protein translation	MHCC97H, Huh7, PLC, MHCCLM3, Hep3B, LO_2_	[28]

### Hypoxia-associated lncRNAs and glucose metabolism

Enhanced aerobic glycolysis is regarded as a hallmark of cancer, which was discovered by Otto Warburg in the late 1920s [44]. In such an environment lacking nutrients and oxygen, cancer cells need to reprogram in glucose to proliferate and survive [45]. The glucose of cancer cells is much more active than normal cells, based on this, the original oxidative phosphorylation needed to switch to aerobic glycolysis to meet the demand of tumors in energy and metabolites [46]. It is known that mTOR and Wnt signal pathways exert a crucial role in glucose metabolic reprogramming and further the function of mTOR to regulate glucose metabolism needs the activation of HIF1α [47, 48].

Recently, studies showed that YAP binds to HIF-1α in the nucleus, sustaining HIF-1α protein stability to bind to PKM2 gene and directly activating PKM2 transcription to accelerate glycolysis under hypoxia stress [23], revealing the significant role of HIF-1 in hypoxia microenvironment of HCC. In addition to PKM2, HIF1α is reported to be able to regulate glucose metabolism by mediating the expression of more than 9 glycolytic enzymes like hexokinase (HK), phosphoglycerate kinase (PGK), glyceraldehyde-3-phosphate dehydrogenase (GAPDH), triosephosphate isomerase 1 (TPI), enolase 1 (ENO1), aldolase (ALD) and so on [49]. The gene coding of HK2 is regulated transcriptionally by HIF-1α, and researchers found a critical eubiquitylase, USP29, could deubiquitylate and stabilize HIF1α, further promoting its transcriptional activity in Sorafenib-resistant HCC patients [50]. In another study, analysis of malignant human liver samples found the intense protein expression of PGK-1 in HCC samples, indicating poor prognosis of HCC patients [51]. Liu et al. discovered that LINC00365 targeted HIF-1α and further decreased the expression of HK2, PKM2, and lactate dehydrogenase A (LDHA) [52], indicating the pivotal role of regulating those key glycolytic enzymes. Collectively, these data revealed that HIF-1α could regulate glycolytic enzymes transcriptionally or by lncRNAs.

Hence, during the hypoxia microenvironment, hypoxia-related lncRNAs are possibly participating in altering glucose metabolism in HCC. On the one hand, hypoxia-inducible NPSR1-AS1 promoted the glycolysis of HCC cells, while HIF-1 regulated it since the researchers observed the expression of NPSR1-AS1 was abolished by knockdown of HIF-1α [53]. Another study suggested silencing of LncRNA RAET1K inhibited increases in lactate concentration and glucose uptake induced by hypoxia. Whereas, HIF-1α upregulates its transcription by bounding to RAET1K promoter region [54]. HIF-1α is increased in various human malignancies including HCC [55, 56], hence, the expressions of those lncRNAs affecting aerobic glycolysis regulated by HIF-1α are also upregulated with any doubt. Combined with the description of hypoxia-associated lncRNAs and metastasis, we can see the multiple role of HIF-1 in HCC, which is indispensable for the malignant progression and poor prognosis in HCC.

On the other hand, lncRNA could also regulate the expression of HIF-1 to affect glycolysis or function independent of HIF-1. The expression of lncRNA homeobox transcript antisense RNA HOTAIR was found to increase in HCC patients' tissues and its knockdown restrained glycolysis in HCC via regulating miR-130a-3p and HIF-1 under hypoxia treatment [24]. Like HOTAIR, lncRNA nuclear receptor subfamily 2 group F member 1 antisense RNA 1 (NR2F1-AS1) was also verified to increase and it was found to regulate HK2 expression by modulating miR-140 [25]. Distinct from the previous two lncRNAs mentioned above, lncRNA LINC01554 was demonstrated downregulated in HCC, exerting as a novel tumor suppressor by promoting the ubiquitin-mediated degradation of pyruvate kinase isozymes M2 (PKM2) and inhibiting Akt/mTOR signaling pathway to abolish aerobic glycolysis in HCC cells [57]. Likewise, lncRNA AC020978 stimulated glycolytic metabolism in non-small cell lung cancer (NSCLC) by directly interacting with PKM2 and enhancing PKM2 protein stability [58]. Seen from this, no matter what kind of cancer PKM2 and HK2 are the common enzymes those lncRNAs target. However, those lncRNAs affecting HIF-1 whether interacting with those lncRNAs regulated by HIF-1 to form a loop are barely investigated and remain elusive. In Table 2, we collected information on hypoxia-associated lncRNAs of metastasis and progression in HCC, presenting the concrete pathway and cell lines.

**Table 2:** Summarized role of hypoxia-associated lncRNAs of glucose metabolism in HCC

	LncRNAs	Expression	Role/pathway	Outcomes	Cell line	References
Glucose metabolism	HOTAIR	Upregulation	Regulating miR-130a-3p/HIF1A	Promoting glycolysis	HepG2 and Huh7	[24]
	NR2F1-AS1	Upregulation	NR2F1-AS1/miR-140/HK2 Axis	Promoting glycolysis and migration	Hep3B, MHCC97-H, Huh7, and SNU-398	[25]
	LINC01554	Downregulation	Downregulating PKM2 Expression and inhibiting Akt/mTOR Signaling Pathway	Mediating glucose metabolism reprogramming	BEL7402, QGY7701, QGY7703, SMMC7721, PLC8024, HepG2, Huh7 and Hep3B	[57]
	NPSR1-AS1	Upregulation	Regulating the MAPK/ERK pathway	Promoting the proliferation and glycolysis	HCCLM3, LO2, HCCLM3	[53]
	RAET1K	Upregulation	HIF1A/lncRNA RAET1K/miR-100-5p axis	Promoting hypoxia-induced glycolysis	HCCLM3, HepG2, Huh7, Hep3B	[54]

### Hypoxia-associated lncRNAs and cancer stem cell-like properties

Cancer stem cells (CSCs) represent a small subset of cancer cells [59, 60] and are capable of initiating tumorigenesis and promoting progression, with aggressive and metastatic features and resistance to chemotherapy and radiotherapy [61, 62]. Similarly, liver cancer stem cells (LCSCs) are also a small subset of cells with unlimited differentiation ability and tumor-forming potential ability [63, 64], owing to this, HCC exerts high postsurgical recurrence rates. Currently, with the rapid advancement of high-throughput sequencing techniques, quantities of lncRNAs linked with the maintenance of cancer stem cell-like properties have been identified [65]. Basic experiments showed that lncDILC inhibited the expansion of LCSCs via mediating interleukin 6 (IL-6)/JAK2/STAT3 pathway and the expression of lncDILC in LCSCs was reduced [66]. LINCR-0003 (lncBRM) is required for the maintenance of the stemness features overexpressed in HCC, and it sustained CSCs properties via YAP1 signaling [66].

However, hypoxia-related lncRNAs regulating CSCs are rare. Recently, a novel lncRNA FERM Domain Containing 6 antisense RNA 1 (FRMD6-AS1) was reported to promote stemness of HCC. The expression of FRMD6-AS1 was increased in tissues and cells in HCC and it was noted that FRMD6-AS1 regulated the protein level of HIF-1α without affecting its mRNA level [29]. Up to now, there are no other reports or studies about hypoxia-related lncRNAs regulating CSCs, so more investigation and experiments are needed to complement.

### Hypoxia-associated lncRNAs and immune escape

The hypoxia microenvironment in tumors makes cancer cells prone to immune resistance phenotype, leading to the occurrence of resistance to immunotherapy [67, 68]. Immune infiltration is an essential factor for the progression of HCC, especially CD8^+^ cells, which can induce the death of tumor cells directly [69]. It is reported that HIF-1α could result in tumor immune escape from CD8^+^ cells by transactivating CD274 and upregulating the expression of PD-L1 [70, 71]. Hence, immune escape and associated cells in the microenvironment exerted an important role in the development and progression of HCC.

Up to now, several lncRNAs have been reported to exert regulatory function in HCC. KCNQ1OT1 is found to combine with miR‑506 competitively and further increase the expression of PD-L1, eventually contributing to the resistance of sorafenib in HCC [72]. Similarly, another study reported that MIAT/miR-411-5p/STAT3/PD-L1 signal pathway may be an underlying therapeutic target for HCC [73]. And lnc-CCNH-8 could upregulate the expression of PD-L1 via miR-217/miR-3173 pathway to induce immune escape in HCC from CD8^+^ T cells [74]. Inferring from this, current research about lncRNAs and immune escape in HCC focuses on the regulation of PD-L1. However, other literatures are demonstrating distinct clues. For example, lncRNA FENDRR is observed to sponge miR-423-5p, upregulate GADD45B, and finally inhibit the immune escape mediated by Treg [75]. On the contrary, LINC00992 has an adverse effect on HCC, which decreases the level of miR-361-5p and increased Twist1 expression, further promoting the metastasis, and invasiveness of HCC [76]. Those results showed the multiple pathways how lncRNAs influencing immune escape. However, research between hypoxia-associated lncRNAs and immune escape in HCC is relatively rare.

There is research investigating the association of the hypoxia-related lncRNA signature with immunotherapy response HCC. Finally, with bioinformatic analysis and validation, five hypoxia-related lncRNAs LINC00869, CAHM, RHPN1-AS1, MKLN1-AS, and DUXAP8 were eventually chosen as the prognostic signature. Those lncRNAs were all relevant to the poor clinical outcomes in HCC with elevated expressions. According to these lncRNAs, the patients were classified as a low-risk group and a high-risk group. The results showed that the low-risk group exerted better prognosis with more abundance in CD8 + T cells and activated B cells than the high-risk group [30], suggesting the viability of these lncRNAs. In line with the bioinformatic results, we can speculate the possibility of hypoxia-related lncRNAs being the early biomarkers and prognostic indicators. In further research, more basic experiments and clinical data are needed to verify the reliability of these predictions, to provide a clinical basis for HCC therapy.

## Current therapeutic advancement

### Assessment of hypoxia level in HCC

HCC was thought to be one of the most hypoxic solid tumors [49]. The normal oxygen partial pressure (pO2) in human tissue is 30 mmHg, while there is only 6 mmHg in liver tumors [77]. Due to this hypoxia microenvironment, the progression, tumor cell proliferation, immune escape, and other aspects are all influenced [11]. More than this, during liver resection, occlusion of blood flow could aggravate the extent of hypoxia. Based on this, monitoring the real-time concentration of oxygen and extent of hypoxia accurately is essential for the diagnosis of the disease and predicting the prognosis of HCC.

In the past decades, various methods to estimate the extent of tumor hypoxia have been explored. First, a kind of nitroreductase-sensitive fluorescent probe was developed and when it directly targeted on tumor, fluorescence could observed with a CRi Maestro spectral fluorescent small animal imager for 90 min [78]. However, this method is invasive and the prolonging time is relatively short. Second, positron emission tomography (PET) can detect the extent of hypoxia in live animals directly when combined with 2-nitroimidazole radiolabeling tracers and computerized tomography (CT) [79]. However, the obstacle in applying PET is the tracers are influenced by hypoxic conditions and other glycolytic byproducts [80]. Thirdly, magnetic resonance imaging (MRI) is a noninvasive evaluation measurement and blood-oxygen-level dependent (BOLD) functional MRI depends on regional differences in blood flow. Whereas, this method is limited by heterogeneous tumor tissue, low regulation of blood flow, and variations in blood vessel size biological factors [81, 82].

In general, accumulating invasive or on-invasive methods are gradually developed, but they are limited in application due to reasons like invasiveness, hypoxic condition influence, and tissue depth. So it is vital to continue developing noninvasive hypoxia imaging measurements to achieve the goal that provides useful information for clinical treatment of HCC.

### Biomarkers of hypoxia-associated lncRNAs

**Fig. 3:**
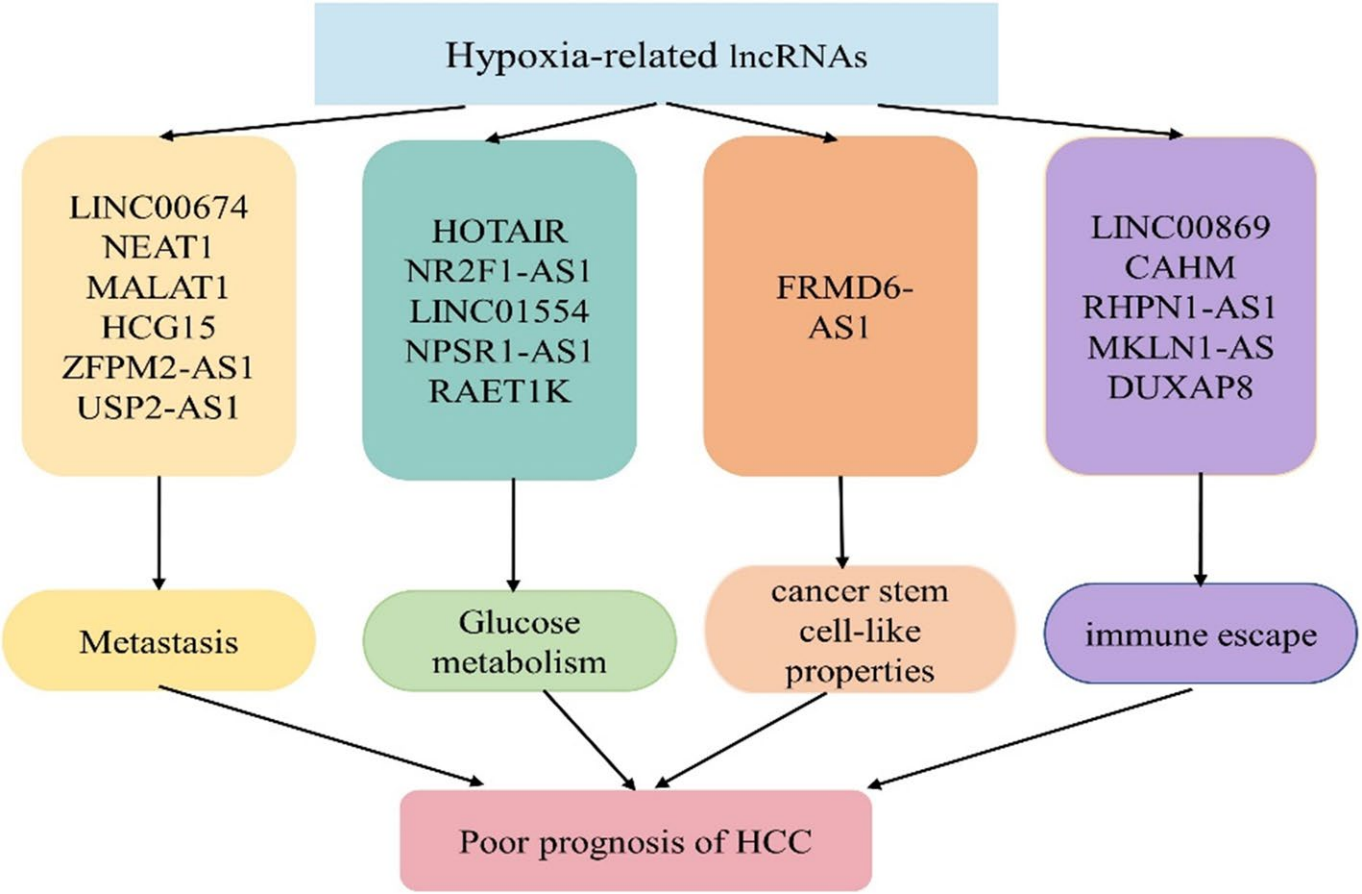
The hypoxia-related lncRNAs affected prognosis and development of HCC via regulating metastasis, glucose metabolism, cancer stem cell-like properties and immune escape. Current reported hypoxia-related lncRNAs in HCC were presented

Current results of researches indicated that compared with normal liver tissue or cells, there is kinds of aberrant lncRNAs expression in HCC [83, 84]. Combined with the fact that lncRNAs are released into plasma or urine [85, 86], which are easy and convenient to assess, lncRNAs possess the possibility being the disease biomarkers to distinguish HCC patients from healthy cohorts. These biomarkers as we mentioned before (Fig. 3), can tell the relevant information about the biological activity of HCC.

We summarized four main types of hypoxia-associated lncRNAs in HCC so that we can tell whether the HCC cells are in proliferation or the process of immune escape. After determining the concrete situation, we could develop a reasonable treatment plan or accurately judge the patient's prognosis. LncRNA NEAT1 was reported as a potential biomarker in digestive system tumors [87], clear cell renal cell carcinoma [88], breast and gynecologic cancers [89], and other cancers, which suggested its viability. However, on the other hand, this may also indicate that it lacks specificity for patients with multiple cancers. Similarly, lncRNA MALAT1 was also reported in various cancers like colorectal cancer [90], osteosarcoma [91], and so on. We have to admit that these hypoxia-related lncRNA are widely aberrantly expressed in various cancers and they do lack specificity for diagnosis of HCC. So more and more investigations and clinical cohorts are urgently needed to solve this problem.

Several studies are reporting combined lncRNAs with recognized HCC biomarker AFP to be a more ideal diagnosis method, rather than applying either one of them [92–94]. For example, the combined application of two lncRNAs UCA1 and WRAP53 with AFP was reported to achieve a sensitivity of up to 100% in a follow-up cohort study of HCC patients [94]. However, there have been no cohort studies about hypoxia-associated lncRNAs up to now, which provided a new direction for future research.

### Clinical application

Current therapy for HCC treatment hasn’t gained satisfactory results owing to high reoccurrence or drug resistance. Hypoxia and its multiple effects also contributed to this situation. In the hypoxia microenvironment, abnormal expression of lncRNAs interacted with various pathways and regulated many signals and proteins to mediate the progression and advancement of HCC. Now the developed lncRNA-targeting approaches are more straightforward and convenient, which contain antisense oligonucleotides (ASOs) and RNA interference (RNAi) technique. These two techniques have exerted good anticancer activities against HCC [95]. Studies have reported that silence of linc00210 by ASO effectively repressed the proliferation and invasion of HCC and downregulation of lncRNA CASC9 via RNAi dramatically decreased the tumor formation [96, 97] By now, mature and commercial experiences of these techniques in HBV application [98, 99] could provide solid basis for therapy of lncRNAs in HCC.

## Summaries and perspectives

Although there is some progress made in lncRNAs in cancers, the concrete function and role of most lncRNAs are still unclear. This is because of their less conserved evolutionarily structure and variable sequence change. In addition to this, there is still a lack of lncRNA knockout animal models, and current experiments in vitro cell lines of human origin can’t compensate for this. An efficient device for detecting hypoxia levels is also needed for further investigation.

The role and function of lncRNAs have gained a lot of attention in research, in which the hypoxia-related lncRNAs are emerging. The essential role of the hypoxia microenvironment and its multiple and complicated interacted pathways are gradually recognized and explored. During hypoxia, the expression of HIF-1 is increased and subsequently, HIF-1 activates the transcription of lncRNAs to affect glucose metabolism, cell proliferation, immune escape, and so on. Most of the hypoxia-related lncRNAs are regulated by HIF-1 via transcription and they are increased in HCC exposed to hypoxia. On the contrary, some hypoxia-induced lncRNAs can also regulate the expression of HIF-1 as its upstream switch or function independent of HIF-1. So the hypoxia-induced lncRNAs still need more data and collection to classify more delicately. The elevated HIF-1 and abnormal expression of lncRNAs contribute equally to the poor prognosis of HCC. Up to now, more and more studies are revealing the underlying hypoxia-related lncRNAs and their associated pathways. There is no doubt that these hypoxia-related lncRNAs are potential biomarkers applying for HCC, but their accuracy and specificity as biomarkers need further validation by basic experiments and clinical cohort studies. RNA vaccines have shown remarkable success, hence, identifying novel hypoxia-related lncRNAs and clarifying their related pathways is beneficial to understanding the unfavorable prognosis of HCC patients, which exerted significant clinical meaning for the early-stage diagnosis and effective treatments of HCC.

## Data Availability

All data related to this paper could be requested from the corresponding authors.
